# Research Progress of Transparent Electrode Materials with Sandwich Structure

**DOI:** 10.3390/ma14154097

**Published:** 2021-07-23

**Authors:** Li-Hao Qin, Yong-Qi Yan, Gan Yu, Zhao-Yi Zhang, Tuofu Zhama, Hui Sun

**Affiliations:** 1 School of Space Science and Physics, Shandong University, Weihai 264209, China; lihaoqin0315@mail.sdu.edu.cn (L.-H.Q.); anxiaoxi321@163.com (Y.-Q.Y.); yugan1610@163.com (G.Y.); jamtuub@foxmail.com (T.Z.); 2 Department of Foreign Language, Qingdao City College, Qingdao 266106, China; zzyqin0212@126.com

**Keywords:** transparent conductive films, sandwich structures, composite electrode

## Abstract

The nonrenewable nature of fossil energy has led to a gradual decrease in reserves. Meanwhile, as society becomes increasingly aware of the severe pollution caused by fossil energy, the demand for clean energy, such as solar energy, is rising. Moreover, in recent years, electronic devices with screens, such as mobile phones and computers, have had increasingly higher requirements for light transmittance. Whether in solar cells or in the display elements of electronic devices, transparent conductive films directly affect the performance of these devices as a cover layer. In this context, the development of transparent electrodes with low sheet resistance and high light transmittance has become one of the most urgent issues in related fields. At the same time, conventional electrodes can no longer meet the needs of some of the current flexible devices. Because of the high sheet resistance, poor light transmittance, and poor bending stability of the conventional tin-doped indium tin oxide conductive film and fluorine-doped tin oxide transparent conductive glass, there is a need to find alternatives with better performance. In this article, the progress of research on transparent electrode materials with sandwich structures and their advantages is reviewed according to the classification of conductive materials to provide reference for research in related fields.

## 1. Introduction

As the nonrenewable and highly polluting nature of fossil energy sources and the relatively high carbon emissions gain more attention from society, the importance of finding efficient and easily accessible clean energy sources is self-evident [1]. Solar energy is undoubtedly a stable and easily available clean energy source, and transparent electrodes play a key role in solar cells [2]. The working part of a solar cell consists of front and rear electrodes and a photoelectric conversion part, with the front and rear electrodes distributed on either side of the photoelectric conversion part. The photoelectric conversion part is based on two main working principles. One is the conversion between light, heat, and electricity, the principle of which is not fundamentally different from thermal power generation. The other is photoelectric power generation, i.e., the photovoltaic effect. Currently, solar cells based on photo-thermal-electric power generation have very limited scope of use because of their high cost. Most of the widely used solar cells are silicon photovoltaic cells, which are based on the photovoltaic effect as the working principle [3]. The front electrode of the solar cell must allow as much light as possible to pass through and to irradiate the photoelectric conversion part, as well as to export the generated current. Therefore, its light transmittance and electrical conductivity must be relatively high to enhance the conversion efficiency of the solar cell. This has given rise to research on transparent electrode materials. In addition, transparent electrodes are required in light-emitting diode (LED)-based display devices [4] where a layer of transparent electrodes is generally arranged on the LEDs, and another layer is arranged under the LEDs to form a conductive circuit. To obtain excellent display effects, the transparent electrodes in LEDs also require good light transmittance and conductivity. The transparent electrodes used in mainstream devices in the market are generally transparent conductive oxide (TCO). In addition, transparent electrode also plays an important role in electrochromic devices [5,6,7].

The development of TCO has been ongoing for more than a century. TCO was first discovered in 1907 by Bakdeker, who prepared transparent CdO conductive films by sputtering and thermal oxidation [8,9]. More than 30 years later, new types of conductive film were made using tin oxide. In contrast, indium oxide thin films and the current mainstream indium tin oxide (ITO) films appeared in the 1970s. Bachmann prepared amorphous and polycrystalline ITO films on different substrates using ion beam deposition and magnetron sputtering in 1978 and applied them to solar cells. Since the 1970s, ITO has been improved continuously. ITO prepared by magnetron sputtering has gradually become the main material for transparent conductive films in various fields [10,11]. However, the limited reserves, high price, and toxicity of indium have limited its large-scale application [12,13]. Moreover, ITO has serious limitations for the large-scale processing of solar cells [14], facing difficulties resulting from high resistivity and poor transparency, and in preparation for LED applications [15]. Aluminum-doped zinc oxide (AZO) is also a material with high transmittance and low sheet resistance. Unlike ITO, AZO does not require expensive raw materials for its preparation. However, studies on doped ZnO show that its performance is generally lower than that of ITO [16,17], while relatively high temperatures are required to improve its performance [18,19].

Because the demand for transparent conductive films has been high owing to the mass production of electronic devices, many researchers have started to seek alternatives to ITO to obtain new transparent electrodes with easily available raw materials and excellent performance. At the same time, to meet the flexible requirements of many new products, such as portable flexible solar panels, emerging folding screen mobile phones, curved display devices [20] and wearable flexible devices [21], electrodes must have good mechanical properties in addition to light transmittance and electrical conductivity, so that the change in resistance during bending is not too large, enabling the device to work properly. Therefore, in recent years, many new electrodes have been designed taking flexibility into consideration. The brittleness of metal oxide ceramic films, mainly represented by ITO and AZO, makes the resistance rise sharply during bending [22] which alone is not suitable for making flexible electrodes. The development of flexible transparent electrodes is, hence, an important direction in the research of transparent electrodes [23,24,25].

Although the conventional single-layer transparent electrode has the advantage of a simple structure, the simple structure makes it difficult to have multiple performance advantages simultaneously. The conventional single-layer transparent electrode is often limited by a certain aspect of its performance, resulting in obvious shortcomings that make it difficult to meet the needs of different applications. In recent years, sandwich structure transparent electrodes have attracted much attention because of their relatively low preparation cost and preparation difficulty (usually prepared using relatively mature methods, such as magnetron sputtering and chemical vapor deposition), their ability to synthesize the advantages of different materials through the synergistic effect of each layer of materials, their superior mechanical properties and flexibility, and their higher light transmittance and electrical conductivity compared with single-layer transparent electrodes [26]. At present, many transparent electrodes have been developed based on sandwich structures with excellent properties, and some of them, such as silver nanowire structure electrodes, have been applied in high-efficiency solar cells [27,28] and LED displays [29]. In addition, since graphene material was invented by Novoselov et al. [30] in 2004, it has been used for the development of transparent electrode materials because of its excellent electrical conductivity and excellent light transmittance. There have also been some studies using such materials as organic materials for the development of transparent electrodes. In this article, the main structures, properties, advantages and disadvantages of sandwich structure transparent electrode materials discovered in recent years are reviewed, and future trends of sandwich structure transparent electrode materials are discussed.

## 2. Sandwich Structure Transparent Electrodes of Different Materials

### 2.1. Metal-Based Composite Electrodes

As conductors, metals tend to have low resistance and good flexibility. Using metals as the materials for composite electrodes can strengthen the mechanical properties of composite electrodes and enhance their electrical conductivity. In general, the light transmittance of a metal is negatively related to its thickness, while the electrical conductivity of a metal is positively related to its thickness. Therefore, to use metal as a component of transparent electrodes, it is necessary to make the metal have a certain thickness that achieves both high light transmittance and low electrical resistance. To meet the requirement of light transmittance, the metal thickness should be controlled at least within 10 nm [31]. At the same time, the thickness cannot be reduced indefinitely because of the difficulty of preparation and the requirement of relatively low resistance. As a result, the main consideration for using metal as the main conductive layer is its structure. Some studies have used thin-layered metal films to ensure that the thickness is not too low and the light transmittance is relatively high, while some other studies utilized metal nanowires or metal nanowebs as the electrodes. This type of structure can ensure that the thickness is not too small, and there is no need to worry about the low light transmission.

**Figure 1:**
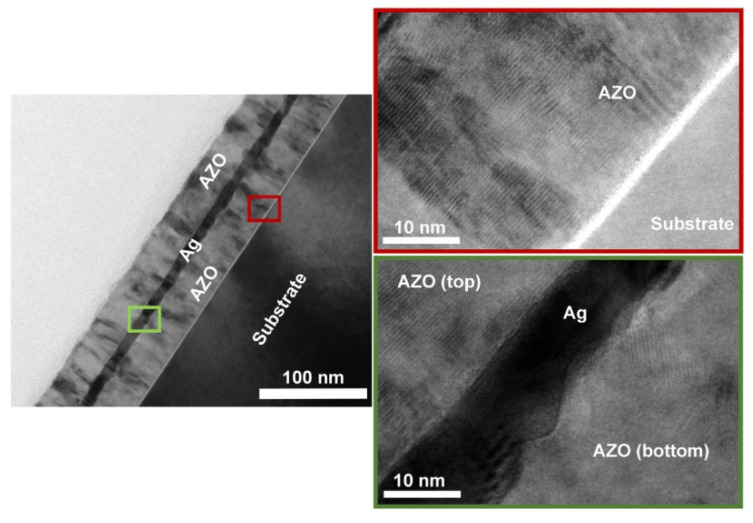
The cross-sectional TEM image of AZO/Ag/AZO [18].

**Figure 2:**
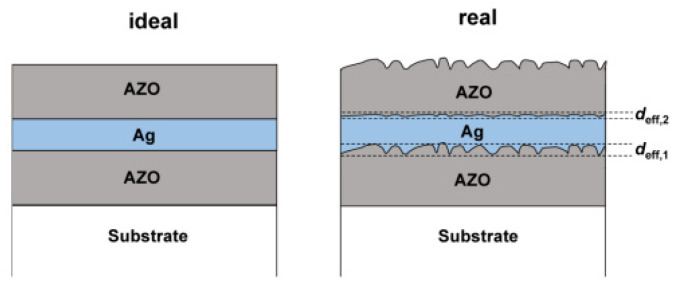
The ideal interfaces and real interfaces [18].

Many recent studies have been carried out based on metal layers as the conducting body. For example, Bingel [18] used the common AZO/Ag/AZO structure. The magnetron sputtering technique was used in an earlier study [32] produced AZO/Ag/AZO films (Figure 1) with thicknesses of 37/10/37 nm, respectively, as well as a sheet resistance of 6.2 Ω/sq and a transmittance of 79.9%. The effect of interface roughness (Figure 2) on transmittance and sheet resistance was also investigated in that study. It was discovered that, as the interface roughness increases, it not only enhances light scattering but also causes the conductivity of the Ag layer to decrease [33] so that the transmittance decreases while the resistivity increases. As a result, the surface roughness is also a nonnegligible factor for the final performance of the electrode material when making composite electrodes. Silver and silver nanowires can be made into metallic films with low resistivity and good mechanical properties [34] which are often used preferentially in electrode materials with similar structures. As mentioned above, as an alternative to ITO, AZO is usually inferior to ITO films in terms of its single-layer optoelectronic properties [16,17]. AZO is also commonly used in composite electrodes to enhance the electrical conductivity while improving the mechanical properties through synergistic effects. In the same way, similar materials, such as ITO and other conventional thin-film electrode materials, can be used in the development of new composite electrode materials by preparing sandwich structures to obtain better performance.

**Figure 3:**
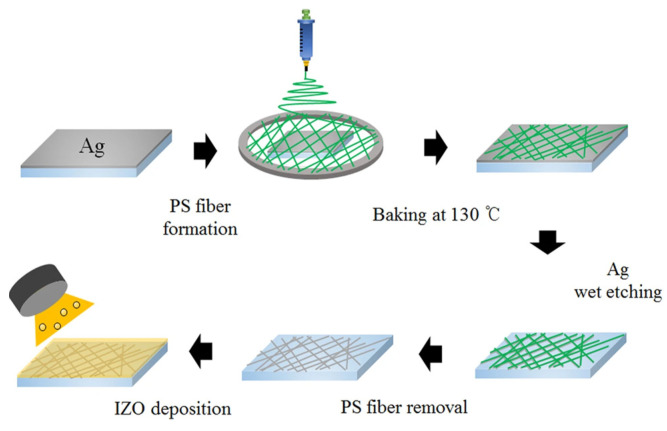
Schematic illustration of the fabrication procedure of Ag fiber/IZO composite electrodes [35].

Choi et al. [35] used a silver fiber solution in their study. The silver fibers were first fabricated by wet etching on polystyrene (PS), which was then covered with IZO films by sputtering to obtain IZO/Ag fiber/PS structured composite electrodes, the preparation flow chart of which is shown in Figure 3. The sheet resistance of the 240 nm-thick Ag fiber/IZO sample prepared in that study was only approximately 10 Ω/sq, which is lower than that of an IZO film of the same thickness (approximately 25 Ω/sq).

**Figure 4:**
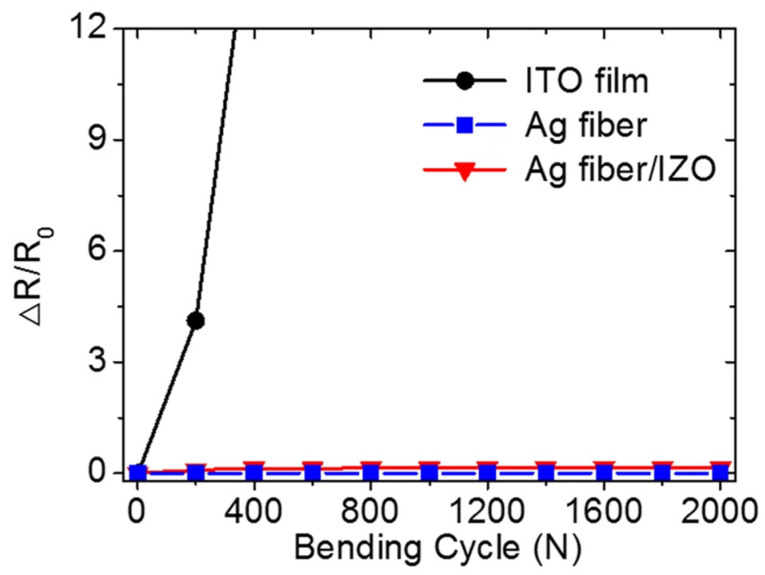
Resistance changes in the cyclic bending test [35].

In terms of transmittance, the transmittance of Ag fiber in the visible light band (400–800 nm) is almost constant at approximately 90%. The light transmittance of the Ag fiber/IZO composite electrode increases from about 50% to greater than 80% in the 400–500 nm interval and fluctuates around 80% in the 500–800 nm interval. It can thus be assumed that the main influence comes from the IZO. However, even though the IZO reduces the transmittance, the transmittance is still better than that of the single-layer IZO film from the control group set up in the study. In addition, the structure has excellent mechanical properties in bending and electrical conductivity. The relative change rate (ΔR/R_0_) for the IZO film exceeds 12 after less than 400 bending cycles with a bending radius of 5 mm, as shown in Figure 4. On the contrary, the relative change rate remains almost zero after 2000 bending cycles for Ag fiber and Ag fiber/IZO composite films. Because of its brittleness, the IZO film has a small curvature to which it can be bent, and it is difficult to ensure that no fracture occurs during the bending process. Once fracture occurs, the conductivity of the IZO film is greatly reduced. In contrast, by adding silver nanowires between the IZO and PS film, the good ductility of the alloy can ensure that not much conductivity is lost during bending. Even if a few Ag fibers break, the conductivity can still be maintained because the large number of Ag fibers in total. At the same time, the IZO and PS film on both sides of the Ag fibers plays a good protective role for the Ag fibers, so that the Ag fibers are protected against external corrosion and abrasion. This significantly improves the mechanical properties while enhancing electrical conductivity and light transmittance; thus, the composite electrode has broader application prospects.

Similarly, You et al. [36] used silver nanowires as the main conducting medium to deposit the two layers of structures, AgNWs and octadecyl trichlorosilane (OTS), on a glass substrate specially treated and formed into a lattice structure. The glass substrate was cleaned and hydrophilized using piranha solution and air plasma; then, the OTS film was deposited on the substrate, and the substrate covered with a quartz chrome mask was etched with UV light to form a grid structure. Finally, a liquid film containing AgNWs, with isopropyl alcohol (IPA) and ethylene glycol (EG) as the solvents, was used to cover and dehumidify the structure to form a composite electrode with a glass/AgNWs/OTS structure containing AgNWs between the lattices. During the dehumidification process, the AgNWs were pulled toward the hydrophilic region because the etched lattice gap was hydrophilic but the OTS remained hydrophobic. Because it is also possible to move AgNWs toward the hydrophilic region indirectly by changing the EG concentration, the EG concentration is also a factor affecting the performance in this study. The innovation of this study is that most electrodes using AgNWs are deposited directly on the substrate in such a manner that the AgNWs are deposited as a random network structure, making the performance difficult to compare with that of ITO thin films. In contrast, the method used in this study can control the radius, length, and orientation of the metal nanowires, which can be combined with other conductive structures in further studies. The test revealed that the performance of the samples prepared showed an overall decrease in both light transmittance and resistivity with increasing deposition rate, as well as an overall increase with increasing EG concentration. The light transmittance was more than 88% in all tests, and the resistivity varied in the range of 20–250 Ω/sq, providing excellent overall performance. The FOM (figure-of-merit) function defined by Sepulveda-Mora [37], a function of the ratio of electrical conductivity to optical conductivity, was used to evaluate the photoelectronic properties of the films as Formula (1) [36]. For transparent electrodes, light transmittance and electrical conductivity often change against one another; thus, this index can better reflect the comprehensive performance of transparent electrodes to a certain extent. Sandwich structure electrodes containing an AgNWs lattice were compared with electrodes containing a randomly precipitated AgNWs structure, and it was concluded that the lattice structure results in an FOM value 13% larger than that of the latter, confirming the superior performance of this structure.

(1) $$T={\left(1+\frac{188.5}{R_{sh}}\frac{\sigma_{OP}}{\sigma_{DC}}\right)}^{-2}$$

Each of the three studies mentioned above has its own characteristics. The conductivity advantage of the metal layer used in the first work leads to the lowest of the three in terms of thickness and sheet resistance but also the lowest of the three in terms of the light transmission, mainly because the metal layer has lower light transmittance than the metal fibers and nanowires [18]. In the second work, silver fibers were employed, which increased the light transmission and sheet resistance slightly, but the thickness increased the most [35]. The third work made some innovations in the preparation route by forming a grid structure on the substrate to control the deposition of silver nanowires, resulting in a grid-like conductive layer, which on the one hand enhances the contact between the silver nanowires to improve the film’s conductivity, and on the other hand minimises the reduction on the light transmittance [36]. At present, the performance of metal-based conductive films is great, as they tend to have a low thickness, sheet resistance and relatively high light transmission, and the metal layer also ensures the ductility of the conductive layer so that there are no significant fluctuations in the film’s conductivity when it is under bending state. Metal layers present excellent electrical conductivity, but their light transmission is disappointed as their thickness increases Thus, the current research prefers to fabricate metal nanowire structures, especially the grid structure as mentioned in the third work, to achieve a good balance between the electrical conductivity and the light transmission. However, the current nanostructures still leave some questions needing to be solved, for example, rough surfaces reducing the light transmission and poor contact between nanostructural layers degrading the conductivity. As a result, in the future work, it may be possible to improve the conductivity and light transmission of the films through optimising the film’s structure to reduce its thickness or through optimizing the deposition process to enhance the contact quality between nanostructures.

When it comes to electrical conductivity, the first thing that comes to mind is metal. Although everyone knows that metal can conduct electricity, no one realized for a long time that most metals can also transmit light when thinner than a certain thickness. The sandwich structure transparent electrode with metal film can obtain good light transmittance while retaining the high conductivity of the metal. Moreover, because of the good extensibility of the metal itself, the new transparent electrode has flexibility which the conventional electrode lacks. The other layers on both sides of the metal also provide good protection for the metal against corrosion and wear while enhancing its durability. It is hence the direction for the development of preparation of transparent electrodes with excellent performance.

### 2.2. Composite Electrodes with Alternating Metal/Compound Materials

When metals are used for composite electrodes, it is necessary to ensure that the thickness of the metal layer is small enough because of the low light transmittance. However, reducing the thickness causes the material resistance to increase, which means that the light transmittance and electrical conductivity are often not optimal at the same time when using metal to prepare composite electrodes. Some metallic compounds can have high optical conductivity with the same thickness. At this time, the thickness can be increased to improve the conductivity, which makes it possible to obtain a higher conductivity with the same light transmittance as metallic materials. In fact, the first to appear in the field of transparent conductive thin film research are metal compounds. In_2_O_3_, SnO_2_, and AZO are typical metal compound electrode materials [38]. They have the advantages of simple preparation and excellent performance, often providing high light transmittance and high conductivity properties in the preparation of composite electrodes.

As mentioned earlier, there are still some difficulties in the study of AgNWs, such as the high surface roughness of coated AgNWs, poor adhesion of AgNWs to the substrate, and thermal instability. To solve these problems, Yu et al. [39] adopted a strategy of combining AgNWs with metal oxides. The AgNWs/ZnO-TF (thin films) were formed by spin-coating ZnO solution on AgNWs-glass substrate and annealing. Subsequently, ZnO-NRs (nanorods) were grown on ZnO-TF by a hydrothermal method, and the ZnO-NRs grew perpendicular to the ZnO-TF layer to finally form the AgNWs/ZnO-TF/ZnO-NR structure. As the ZnO-NR continued to grow, its light transmittance decreased gradually, while the haze increased gradually. After 1 h of growth, the transmittance increased from 60% to 80% with a wavelength of 400–500 nm and then remained slightly below 80%, while the haze was less than 20% in the visible wavelength range. After 3 h of growth, the transmittance was slightly lower than that after 1 h with a wavelength of 400–700 nm, which then remained essentially the same. The haze decreased from nearly 100% to approximately 40% with a wavelength of 400–600 nm, followed by small fluctuations in the range of 20–30%. This structure was used for the first time in this study, enhancing the adhesion of AgNWs to the substrate to some extent and making it possible to control such properties as transmittance and haze by controlling the growth time of the ZnO-NR. However, the upper limit of its transmittance is low, with an average value of less than 80%, and the study did not clearly reflect the change of the sheet resistance of this structure as a transparent electrode. This perhaps can be elaborated in subsequent studies. However, the application of this ZnO-NR structure provides some new properties, such as enhanced substrate adhesion and formation of a three-dimensional optical trap structure, and it is possible to change the optical properties of the ZnO-NR structure to some extent by changing the growth time (essentially the spatial density of ZnO-NR). This can serve as a reference for the preparation of materials of the same structure.

In a study by Morita et al. [40], AGZO films with one to five layers of ZnO doped with both Al and Ga were prepared by spin coating as well as annealing. The substrates were cleaned by a special treatment method called the “SC-1 method”, which uses an SC-1 solution containing ammonia, hydrogen peroxide, and pure water. The SC-1 solution was ultrasonically cleaned at 80 °C and rinsed with pure water to achieve a better rinsing effect on organic matter. When the team prepared a single layer of AGZO with a thickness of approximately 60–80 nm, it was found that the AGZO layer was discontinuous and the glass substrate was exposed, resulting in a significant impact on both conductivity and light transmittance. The team then prepared a second layer of AGZO by spin-coating and annealing the AGZO precursor on the first layer and found that the discontinuities were improved. Therefore, the team used this method to fabricate AGZO structures of one to five layers and compared them. Based on this method, for the three-layer AGZO film structure, light transmittance with a 400–700 nm wavelength was consistently in the range of 90–100%, while the sheet resistance was approximately 2.5 MΩ, substantially lower than that of the one-layer and two-layer structures. The large number of discontinuities in the one-layer and two-layer structures resulted from structural defects, such as partial breaks or uneven thicknesses within the layers, which increase the light scattering and sheet resistance. The three-layer structure had almost the same number of discontinuities as the four- and five-layer structures, which indicates that the three-layer structure eliminated most of the discontinuities. The three-layer structure had low sheet resistance and better transmittance than the four- and five-layer structures, achieving an optimal overall performance that supports the advantages in the performance of the sandwich structure.

**Figure 5:**
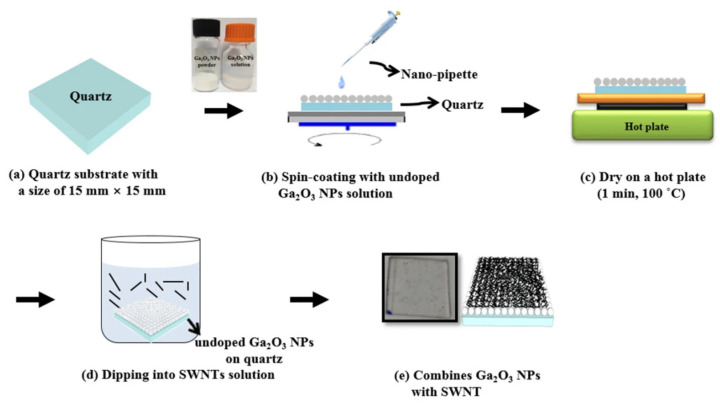
The schematic illustration for spin and dip-coating procedure of proposed Ga_2_O_3_-NP/SWNT layer on quartzes [41].

**Figure 6:**
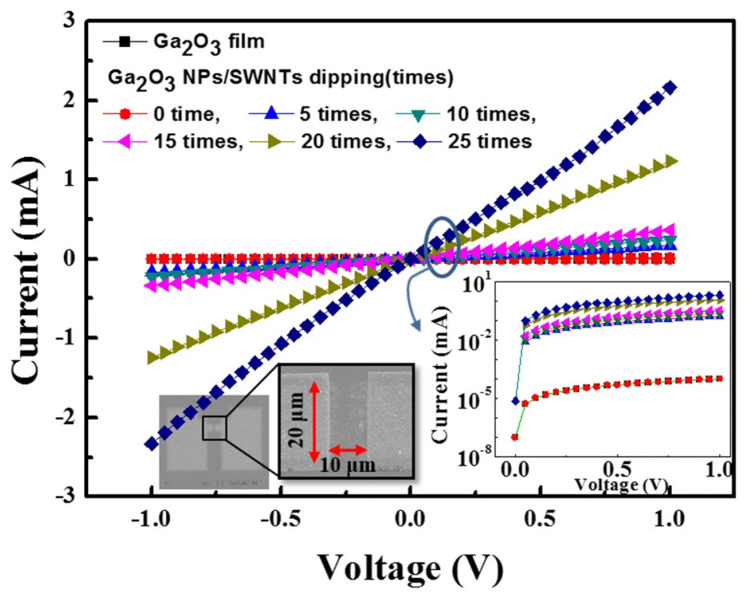
Current–voltage characteristic curves [41].

**Figure 7:**
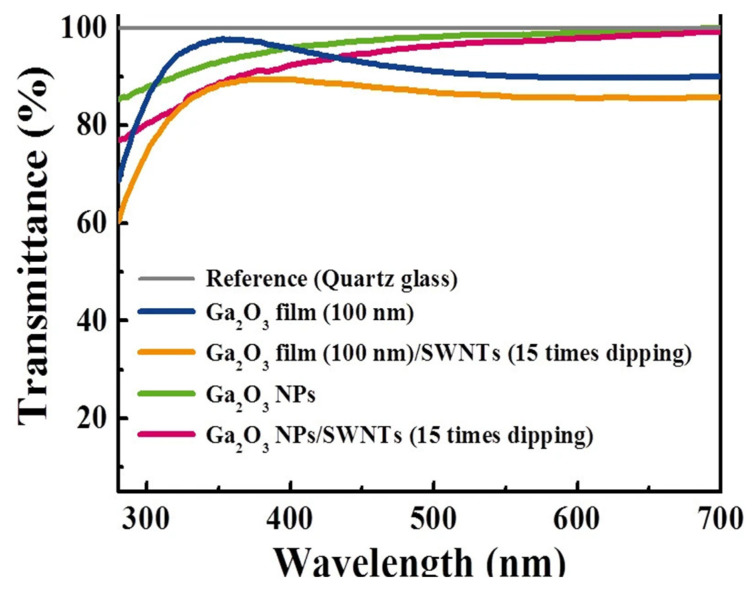
Optical transmittance spectra of undoped Ga_2_O_3_ film [41].

In a study by Kim et al. [41] a two-layer structure was deposited on a quartz substrate, resulting in a single-walled carbon nanotube (SWNT)/gallium oxide nanoparticle (Ga_2_O_3_-NP)/quartz substrate structure. The undoped Ga_2_O_3_-NP solution was first spin-coated on the quartz substrate, which was dried, immersed in SWNT, and dried in nitrogen (Figure 5). The I–V curve (Figure 6) and light transmittance (Figure 7) of the SWNT/Ga_2_O_3_-NP/quartz were measured in subsequent tests in the study. The addition of SWNT caused the SWNT/Ga_2_O_3_-NP/quartz structure to exhibit a lower resistance than the stand-alone Ga_2_O_3_ film, and the resistance gradually decreased with increasing immersion time in the SWNT. The light transmittance was slightly lower than that of the one-layer Ga_2_O_3_-NP for the SWNT/Ga_2_O_3_-NP/quartz structure because of the introduction of the SWNT, but the composite structure still outperformed the other two control structures shown in in Figure 7 without the Ga_2_O_3_-NP layer (i.e., Ga_2_O_3_ film and Ga_2_O_3_ film/SWNT). The transmittance was basically higher than 80% with a wavelength of 300–400 nm and was between 90% and 100% with a wavelength of more than 400 nm. However, the resistance of the resultant composite structure was still much higher than that of commercial ITO films; however, the problem may be solved by doping the Ga_2_O_3_-NP.

The above-mentioned works related to the new structures or novel compositions. Although the low sheet resistance and the high light transmission can still not be well balanced in these works, there are still some points can be ported to the future work. For example, the ZnO-NR structure electrode in the first study of this section can be used as an electron transport layer in solar cells and can increase the efficiency of photovoltaic conversion [39]. In the third study, the combination of carbon nanotubes and Ga_2_O_3_ nanostructures could achieve excellent light transmission. Future work in this direction should focus on simultaneously enhancing the electrical properties and optical properties of these kind of transparent conductive films in order to expand their applications [41].

Metal compounds include the typical metal oxides. Some metal compounds have electrical conductivity and light transmission to a certain extent simultaneously, making it possible to prepare thicker composite electrodes. However, in recent years, there have not been many composite electrodes based on metal compounds with new compositions or structures. There are two possible reasons for this. On the one hand, conventional electrodes of the same type based on such materials as ITO and AZO are very mature; on the other hand, metal compounds do not have an advantage in terms of conductivity, which is the most important property of electrodes. In addition, it is difficult to achieve a greater advantage in light transmittance at the same time, because the mature electrodes themselves already have high light transmittance. Nevertheless, some new worthy structure ideas are being adopted and developed, possibly leading to the emergence of more excellent electrodes.

### 2.3. Carbon-Based Composite Electrodes

At present, the most representative and promising carbon-based material is graphene. Since its invention in 2004, graphene has been favored in many fields because of its excellent thermal conductivity, electrical conductivity, mechanical properties, and light transmittance. Because of its excellent electrical properties and light transmittance [42,43] graphene has also become a promising new transparent electrode material. However, the sheet resistance is large when using only graphene films [44]. As a result, using graphene as part of the composite electrodes has become an option to improve the performance of the composite electrodes. In addition, the excellent mechanical properties of graphene [45] can make it extremely useful in the application of flexible transparent electrodes.

Liu et al. [46] developed a multilayer graphene/graphene scrolls (MGGs) structure by first arranging the layers in the order of poly methyl methacrylate (PMMA), graphene, gold (Au), and graphene. The Au layer was then etched, such that the graphene layer not attached to PMMA on one side of the Au layer became rolled up into scrolls because of surface tension. The same procedure was repeated for the graphene layer attached to PMMA to obtain the graphene-graphene scrolls repeating structure. The multilayer repeating structures can enhance the strain tolerance. At 100% vertical strain, the resistance of the three-layer MGGs structure increased by only 50%, while the resistance of the control three-layer graphene without graphene scrolls increased by 300% under the same test conditions. The data in this study show that the resistance is in the range of 10^2^–10^3^ Ω/sq when the light transmittance at a wavelength of 550 nm of the multilayer graphene–graphene scrolls is in a range of approximately 87–97%. The mechanical properties of the three-layer graphene–graphene scrolls structure are also excellent, with only an eight-fold increase in resistance at 1000 strain cycles compared with an approximately 100-fold increase in the control. When a graphene layer breaks under bending state, the graphene scrolls can also act as a conductive structure between the cracks. Due to its long length, the graphene scrolls can usually bridge cracks of micron width, thus avoiding the deterioration in sheet resistance. This is also the common strategy used by most flexible electrodes to solve the problem of flexible conductivity. Sometimes in multi-layer structures there is a situation where a membrane layer is not flexible or is not flexible at all. In this case, the adjacent flexible layer will bridge the cracks in the less flexible layer, and the non-adjacent flexible layer will continue to perform its electrical conductivity so that the overall electrical conductivity of the films does not change significantly.

**Figure 8:**
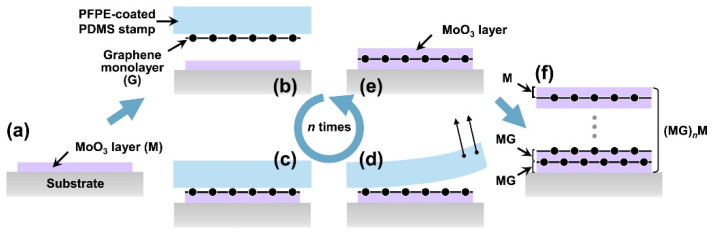
Schematic of the fabrication of MoO_3_-interlayer-doped graphene multilayers [47]. Thermally deposited a MoO_3_ layer on a substrate (**a**). A graphene monolayer deposited on a PFPE-coated PDMS stamp by a modified wet-transfer method is transferred onto the MoO_3_ layer by a transfer-printing process [(**b**–**d**)]. Next, another MoO_3_ layer is deposited on the transfer-printed graphene layer (**e**). By performing steps (**b**) through (**e**) n times, (MG)nM is obtained (**f**).

Cho et al. [47], however, used a MoO_3_/graphene/MoO_3_ structure (Figure 8). Because MoO_3_ has a very high work function [48], p-doping of graphene was achieved by inducing efficient transfer of electrons from graphene to MoO_3_ [48,49]. The graphene film was deposited on the copper film by chemical vapor deposition, which was subsequently transferred to polydimethylsiloxane and then onto the MoO_3_ layer. The process was repeated to obtain the MoO_3_/graphene/MoO_3_ structure. The test data reveal that with (MoO_3_/graphene)n/MoO_3_, i.e., the (MG)nM structure with n = 5, the light transmittance was still more than 85%, and the sheet resistance was approximately 200 Ω/sq. The team also studied (graphene)n/MoO_3_, i.e., the GnM structure. It was revealed that the GnM structure had a small increase in light transmittance (~1.5–3%) and an increase in sheet resistance of 150–300 Ω/sq. The overall performance was inferior to that of the (MG)nM structure. In a subsequent mechanical test using (MG)3M electrodes on polyethylene terephthalate (PET) substrates, the relative rate of change in the sheet resistance of the sample remained almost constant at approximately 0% for 1000 repetitions of bending with a 10 mm radius of curvature and 1% strain. In contrast, even bending tests performed with a 5 mm radius of curvature and 2% strain resulted in a relative rate of change in the range of only 3–7%. The thermal evaporation and transfer printing processes used in this study may inhibit the creation and expansion of cracks within the layer.

**Figure 9:**
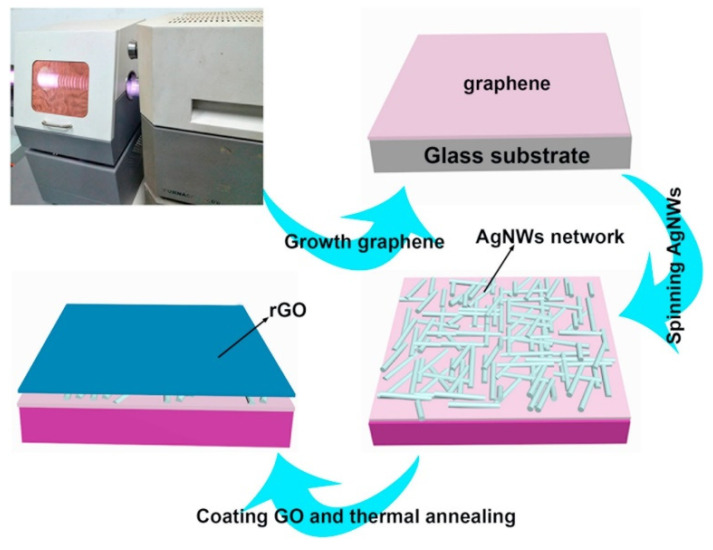
Schematic of the fabrication process of the G/Ag NWs/G sandwich film on a glass substrate [50].

**Figure 10:**
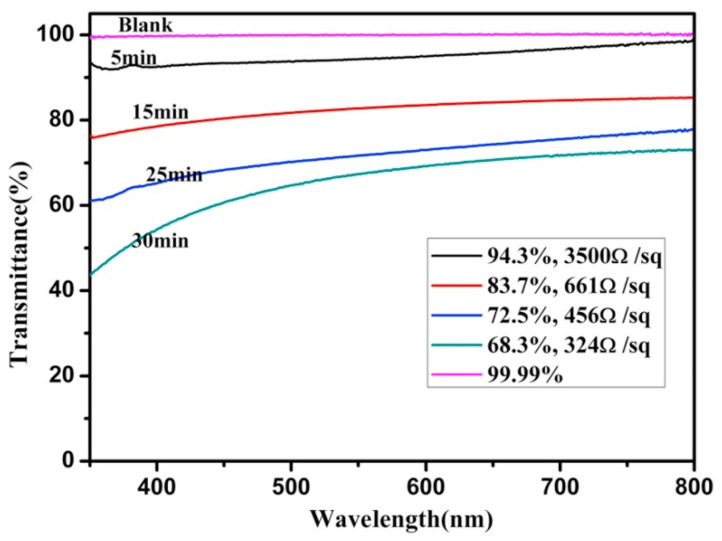
UV–VIS spectra of graphene films grown on glass with varying growth time (5, 15, 25, and 30 min) and the corresponding sheet resistance [50].

**Figure 11:**
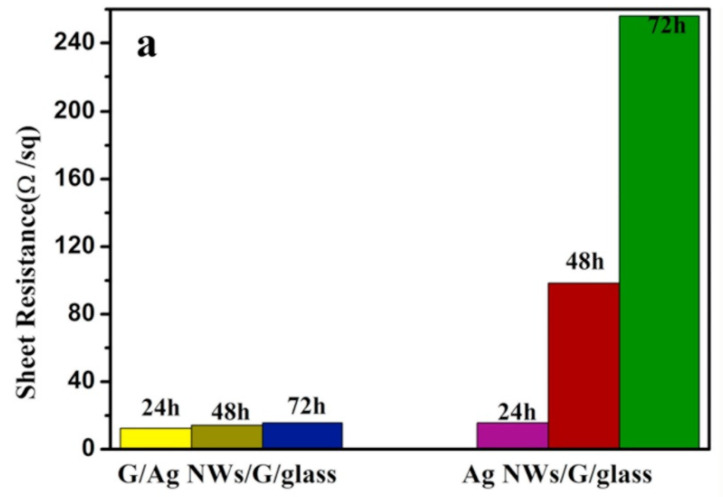
Changes of sheet resistance of G/AgNWs/G/glass and AgNWs/G/glass after exposure to air at 95 °C for 24, 48, and 72 h [50]. The original figure is from reference [50], figure 11 (a).

The above two studies show that, although the light transmittance of the composite electrodes is quite good, the sheet resistance is on the order of 102 Ω/sq for the smallest value and 103 Ω/sq for the largest value. This resistance is still high for an electrode, which inevitably leads to unsatisfactory product performance in practical applications or the heating of the electrode material over a long period of time. Combining graphene with the metal layer introduced in the first part of this review may lead to better sheet resistance. The structure developed by Chen et al. [50] used a combination of graphene/AgNWs/graphene (G/AgNWs/G) to obtain a lower sheet resistance. Plasma-enhanced chemical vapor deposition was first used to generate graphene films on the glass substrate, and AgNWs was spin-coated on the first graphene film, followed by spin-coating graphene oxide solution twice. The film was dried at room temperature and annealed for 20 min to enhance the adhesion between the graphene and AgNWs films (Figure 9). Figure 10 shows the light transmittance of the graphene layer and the corresponding sheet resistance with time during the growth of the graphene film on the glass substrate. In the study, for the stand-alone graphene film, the light transmittance is high, and the sheet resistance is also high after 5 min of deposition. The light transmittance drops considerably after 30 min of deposition, but the sheet resistance is not low still. Figure 11 shows the sheet resistance of the final prepared structures, such as G/AgNWs/G. The sheet resistance of the G/AgNWs/G structure is always less than 20 Ω/sq. According to the study, the final prepared sample has a light transmittance of 77.6% and a sheet resistance of 10.8 Ω/sq. The sheet resistance only increased by 3.8 Ω/sq after 72 h at 95 °C. Comparing these data with the data shown in Figure 10, one can see that the addition of the AgNWs structure played a crucial role in reducing the sheet resistance from an initial order of magnitude between 102 and 103 Ω/sq to the order of 10 Ω/sq, while maintaining a relatively good transmittance. Figure 11 shows that the stability of the G/AgNWs/G structure has been improved in the high-temperature environment compared with that of the AgNWs/G structure. This to some extent indicates that, when graphene instead of the AgNWs structure is exposed to a high-temperature environment, the better chemical stability and heat resistance of graphene can lead to better protection for the nanostructure. In addition, the better thermal conductivity of graphene enables the electrode material of this structure to be used for heat dissipation systems in specific application scenarios, to obtain higher operating currents, etc. Mixing graphene materials in several other types of composite electrode mentioned in this review would further enhance their structural stability, conductivity and light transmittance; thus, this is a worthwhile area for further research.

The films prepared in these three works in this section all perform well, with both low sheet resistance and high light transmission. The pure graphene electrode used in the first study has excellent light transmission and mechanical properties. The second group has a thin thickness comparable to that of electrodes using layered metals. The third group combines the advantages of graphene and metal nanowires; although slightly lower in light transmittance, the sheet resistance in this study is to the most closed to that of the metal layer electrodes. Graphene-containing transparent conductive films are full of potential.

As a focus of research in recent years, graphene is indispensable in the field of transparent electrodes. While the sheet resistance of graphene is not ideal when it is used alone, its comprehensive performance can be enhanced when combined with metals. For example, the mechanical properties of graphene enable it to provide good protection for the nanostructures within the coating, and further enhancing the mechanical properties of the electrode can meet the needs of gradually developing flexible electronic devices. There is still considerable room for the development of graphene in this field with huge application prospects. Combining graphene with other structures discussed in this review might lead to the development of new electrodes with amazing performance, which is a topic worthy of further research and development.

### 2.4. Organic-Dominated Composite Electrodes

Organic materials are often neglected in the research field of transparent electrode conductivity, with most studies focusing on inorganic metal or nonmetal materials. Nevertheless, many organic materials have driven technological progress to some extent [51] and some of them have high light transmittance and conductivity to a certain degree, such as PEDOT:PSS [52]. Therefore, organic materials can also be applied in the preparation of transparent conductive films. Moreover, organic materials tend to have excellent flexibility, which can enhance the mechanical properties of the electrodes [53,54].

Kim et al. [55] used organic materials, which have a single purpose with a significant effect. Nylon 6 nanofibers were used for the sole purpose of enhancing the mechanical properties of the transparent composite electrodes. In their study, nylon 6 nanofibers were first deposited on ITO films using the solution electrospinning method, followed by filling the interstices between the nanofibers with cellulose acetate solution by a solution casting method and subsequently spin-coating AgNWs on the film. In the study, the bonding layer of nylon 6 nanofibers and cellulose acetate was referred to as “NF-r-CA”, resulting in an “ITO/NF-r-CA/AgNWs” structure. Both ITO and AgNWs are commonly used materials in the field of composite electrodes. The NF-r-CA film cannot conduct electricity and is designed specifically to enhance the mechanical properties of composite electrodes. The design is also a good illustration of the concept of composite electrode design. In a multilayer structure, each layer has its own advantages and strengths as an individual; at the same time, the overall performance is enhanced by the synergistic effect of the multiple layers. In the NF-r-CA film, the characteristics of the nylon 6 nanofibers are exploited to the maximum, and their mechanical properties are greatly increased with the increase of nanofiber content. Forty-five minutes of electrospinning can result in a NF-r-CA film with a tensile strength of 59.7 MPa and a toughness of 586 kN/mm, which is approximately nine times more tough than CA film alone. The CA component also fills the gaps between the NFs, enhancing the surface smoothness, reducing light scattering, and improving the light transmittance, which did not fall below 90%, even after 45 min of electrospinning. The team finally selected the sample with 45 min of electrospinning, which has better overall performance as the raw material for the composite electrode. Because of the excellent performance of NF-r-CA, the final electrode showed excellent performance, with a stable light transmittance of approximately 90% in the visible band and a sheet resistance of only 24 Ω/sq. In terms of mechanical properties, the relative rate of change of the sheet resistance of the commercial ITO electrode approached 300 with a bending radius of 1 mm, which also increased linearly after more than 250 bending cycles in the 1-mm repeated bending test until the electrical signal was lost. In contrast, the relative rate of change of sheet resistance of the ITO/NF-r-CA/AgNWs electrode was almost constant when the bending radius was decreased from 10 to 1 mm, and it only changed 0.89% in 10,000 bending cycles, which is remarkable.

In a study by Singh et al. [56], organic materials were combined with ITO films to prepare a transparent flexible conductive film with high light transmittance and high electrical conductivity along with excellent bending and stretching properties. In the study, a two-layer structure was deposited on a PET substrate. A layer of the hydrophilic 3-aminopropyltriethoxysilane was first prepared on the PET substrate using molecular vapor deposition, a buffer layer of PEDOT:PSS solution approximately 50 nm thick was then spin-coated, and a layer of ITO of approximately 100 nm in thickness was prepared on it by magnetron sputtering, resulting in an ITO/PEDOT:PSS/PET (IPP) structure. According to the test results, the transmittance of the composite electrode rapidly increased from slightly more than 30% to 60% at a wavelength of 300–350 nm and then smoothly increased to slightly less than 90% with a wavelength in the range of 350–600 nm. Although its light transmittance was approximately 10% lower for wavelength in the range of 600–800 nm compared with the ITO alone, this is still acceptable. The sample haze was always less than 2%, indicating that the clarity of the sample was high. The sheet resistance of the IPP film was around 40–60 Ω/sq. The main goal of the study was to improve the mechanical properties. For the PEDOT:PSS/PET structure, the sheet resistance was always on the order of 102–103 Ω/sq at a maximum strain in the range of 0–0.35. For the ITO/PET structure, the sheet resistance reached 105 Ω/sq at a maximum strain of 0.10, which further rose linearly to 108 Ω/sq when the maximum strain was increased to 0.15. In contrast, for the IPP structure, the sheet resistance increased from less than 102 Ω/sq to approximately 103 Ω/sq when the maximum strain was in the range of 0–0.025, which then gently increased to slightly below 105 Ω/sq when the maximum strain is in the range of 0.025–0.35. This shows a significant improvement in mechanical properties compared with ITO, in large part because the PEDOT:PSS structure still functions as a conductor, preventing the overall resistance from increasing too rapidly. In addition, because of the coverage of ITO, the thermal stability of the structure was somewhat improved, with the average sheet resistance changing by not more than 5% at 80% ambient humidity and 50 °C. Nevertheless, its thermal stability still must be improved as a polymer because heat generation is inevitable when electronic devices are used for a long time. At the same time, the resistance of the electrode material rises after bending, which leads to further heat generation. Because 50 °C is not considered high temperature in the use of electronic devices, the stability at 50 °C is not as convincing, and how to maintain its stability at a higher temperature or in an environment at a higher temperature for a long time is a problem that still needs to be solved. Furthermore, the PEDOT:PSS polymer is not stable enough in the atmosphere [57], possibly leading to difficulties for the preparation and long-term use of electrodes with this structure.

The number of studies using polymers as one of the raw materials to make transparent electrodes is relatively small compared with studies of the other structures mentioned in the references above. Moreover, the performances of some of the few structures prepared are not very good. Nevertheless, there are still some structures that are worth studying, and several of the above studies show that certain polymers with outstanding physical properties lead to a relatively large improvement in the overall performance of the corresponding composite electrodes. Future research could be devoted to compensating for the shortcomings in the physical properties of these polymers through other structures, such as the enhancement of mechanical properties by the NF-r-CA layer in the first study and the maintenance of electrical conductivity after bending by the PEDOT:PSS layer in the second study [55,56].

## 3. Comparison and Outlook

Here we have summarized the thickness, sheet resistance, and transmittance of some above introduced film electrodes (Table 1). In terms of thickness, some of the experiments were conducted using silver nanowires or other structures that resulted in a rough surface, so that only an approximate thickness range could be estimated. Other experiments were using metal or oxide films that were easier to obtain smooth surfaces, so that more accurate thickness values could be measured. It can be seen that the thicknesses of the materials are all in the order of 10^1^–10^2^ nm, which suggests that the thickness of transparent conductive films with good overall performance should be at least within this order of magnitude. Given the limitations of the current preparation technology, too thin film generally possesses higher sheet resistance, and too thick film usually has a significant reduction in light transmission.

The performance of each material in terms of sheet resistance varies. The lowest sheet resistance is the AZO/Ag/AZO structure with only 6.2 ohm/sq, and the highest sheet resistance is the triple AGZO structure with 2.5 Mohm/sq. The conductivity of transparent conductive films is of paramount importance, and high sheet resistance will undoubtedly affect performance in applications where low sheet resistance means the ability to conduct higher currents with less current thermal effects. In the section on sheet resistance, it can be noted that studies using metal or metal nanowires tend to have low sheet resistance, mostly in the order of 1–10 ohm/sq. The only exception is the study of AgNWs/ZnO-TF/ZnO-NR structures with their sample sheet resistance reference values above 100 ohm/sq. It should also be noted that when using the current mainstream four-probe method to measure sheet resistance, if the silver nanowires are on the surface, their actual conductivity should be higher than the measured value. It is not difficult to understand this theory, i.e., assuming that there are two sets of studies with silver nanowires and silver layers on the surface and that they have the same measured sheet resistance, it is clear that the contact area of the silver nanowires in contact with the probe will be smaller than that of a normal layer structure and that the former will tend to outperform the latter in terms of actual conductivity. Sheet resistance was on average higher in those studies that did not use metals or metal nanowires, a result that is not surprising considering that metals are still the first choice for conductivity, but some of the studies related to non-metallic materials also exhibited a low sheet resistance. As in the two studies with graphene and no AgNWs, the sheet resistance is in the range of 100–200 ohm/sq, while the ITO/PEDOT:PSS/PET structure achieves an even better 40–60 ohm/sq. In general, the current research prefers to combine metals with metal oxides or non-metallic materials in order to achieve low sheet resistance, but at the same time, some completely non-metallic electrode structures also have good sheet resistance, which shows us the potential and value of research in this direction. Perhaps in the future, non-metallic transparent conductive films can also achieve low sheet resistance comparable to that of metal-containing electrodes.

In the third section, most of the studies have light transmittance in the 80–90% range. The light transmittance levels of carbon-based transparent conductive films are slightly higher than the other types of studies, and the light transmittance of organic-based related studies fluctuates up and down. However, in general, the light transmittance of these studies is good enough for relevant devices such as solar cells or display devices.

In terms of material flexibility, both metal-based, carbon-based and organic-dominated materials show good performance. The carbon-based and some organic matter-based electrodes perform very well. Most metal oxides are difficult to ensure flexibility due to their brittle nature and often require the use of nanostructures or distributed structures to increase their flexibility.

The more commonly used transparent electrode preparation processes are magnetron sputtering, spin coating (often accompanied by annealing), wet etching, solution electrostatic spinning, MVD, and other methods. Metal-based and metal-compound-based transparent electrodes are predominantly used by magnetron sputtering, wet etching, and spin-coating. Spin-coating is mainly applied to the deposition of nanostructures such as silver nanowires and gallium oxide nanoparticles. The preparation of carbon-based and organic-dominated materials is more variable and often depends on the specific material and the specific structure. Examples include the CVD and PECVD methods used for depositing graphene, the preparation of graphene scrolls at the expense of metals, the preparation of organic matter electrodes by the MVD method and spin coating, etc. Some commonly used methods of preparation are not covered in this study, such as molecular beam epitaxy and vacuum vapor deposition. The magnetron sputtering method is highly efficient and produces good quality films, but the preparation temperature is often high and is suitable for metals and metal compound materials with good heat resistance. Spin coating is simple and inexpensive, but the nanostructures deposited by it are often randomly distributed, making it difficult to guarantee good contact between the nanostructures and requiring additional methods to be devised to regulate the distribution of the nanostructures. Among the mainstream methods of graphene preparation, CVD is more suitable for the preparation of thin film electrode materials. Depending on the structure and purpose of each study, there will be significant variability in the subsequent preparation methods. As organic materials are usually not resistant to high temperatures, low temperature preparation methods are often preferred in order to avoid damaging the structures and compositions of the organic materials.

In the preparation process, as the preparation of flat film layers often used in magnetron sputtering or spin coating and other methods for the cleanliness of the substrate required high standard, so first before the preparation can choose the appropriate substrate cleaning methods, such as the use of piranha solution, air plasma, the use of ultrasonic cleaning, the use of pure water for rinsing, etc., which will better ensure the cleanliness of the substrate surface. A smooth and flat film layer has a significant improvement in light transmittance. During the preparation process, nanostructures are usually prepared by spin-coating, in which multiple spin-coating and annealing can be carried out. The multiple spin-coating is done to reduce or avoid cracking of the film layers, while the annealing enhances the adhesion between the layers. The nanostructures should not be placed on the surface layer, as more wear will result in poorer contact between the nanostructures, but in the middle layer, where they can be protected by the film on both sides. The nanostructures should not be placed on the surface layer, as more wear will result in poorer contact between the nanostructures, but in the middle layer, they are protected by the film on both sides, thus improving the durability of the electrodes. Secondly, after the nanostructures have been prepared, as they are not spatially continuous, a small amount of filling in the gaps between the nanostructures may be considered to enhance the surface flatness and filling with a conductive medium may also enhance their electrical conductivity. In the preparation of spatially structured film layers, such as the aforementioned ZnO-NR and graphene spools, the choice or innovation of preparation method needs to take into account the actual situation of the target structure. For example, graphene scrolls are prepared using a sacrificial metal layer, which allows the graphene layer to be rolled up into a scroll by surface tension. Finally, the layers can also be arranged to achieve the tightest possible bond, taking into account the difference in adhesion between the different materials.

## 4. Conclusions

In recent years, sandwich-structure-based multilayer films have been selected to make transparent conductive films. In this review, four main research directions different from the conventional transparent conductive films based on the different selection of materials were introduced. First, as the most common conductive medium, metal is used the most frequently in the field of sandwich structure transparent conductive films, whose applications are also the most mature. The main design ideas used are to control the thickness of the metal so that it obtains a certain light transmittance while retaining good conductivity or to use nanofiber structures to maintain the light transmittance of the film. However, the metal film thickness is limited by its light transmittance. The fabrication of ultrathin metal films with smooth surfaces and no faults requires advanced processing technology, which also limits the reproducibility of the electrode. Moreover, the fabrication of nanostructure metal layers is limited by the high production cost [58]. Therefore, for the preparation of metal composite electrodes, the main challenges are the strict control of the metal layer thickness, obtaining good reproducibility, and reducing the production cost. Second, it is difficult for the composite electrodes with new metal compounds to surpass the conventional thin films made of ITO or FTO, and there are not many novel structures and properties found in the relevant research. Third, as an intensively studied topic in recent years, graphene has played many positive roles in composite electrodes because of its light transmittance, mechanical properties, and electrical conductivity, making graphene an important research direction, which should be expanded for use in other fields. Finally, polymers have surprised many with their excellent performance in some specific aspects—for example, their excellent mechanical properties can greatly enhance the flexibility of electrodes. However, there is little gain in electrical conductivity, and their thermal stability and durability must be strengthened. This direction has great potential but still needs significant effort to develop.

The key point in the field of sandwich structure transparent conductive films is the composite structure with the multiple layers of films. As mentioned earlier in this review, the principle that the multilayer film structure needs to follow is such that each layer should have its own independent characteristics (strong electrical conductivity, good light transmittance, excellent mechanical properties, etc.) At the same time, as a whole, they must influence the overall structure with their respective advantages through the synergistic effect between the materials. The number of research studies in this field is still small, including in some more mature research directions, and some research is still less worthy of further development. The future research should focus on the diversification of the structure with the existing materials, such as the use of a silver nanofiber structure. Meanwhile, it is worth trying to combine different types of material in multiple ways, such as composite electrodes made of graphene, metal, and organic materials, to combine the strengths of the materials better and enhance the comprehensive performance of the composite electrode.

## Figures and Tables

**Table 1:** Summary of the thickness, sheet resistance, and light transmission of some studies.

Material	Thickness/nm	Sheet Resistance/Ω·sq^−1^	Transmittance	References
AZO/Ag/AZO	37/10/37	6.2	79.9%	[18]
IZO/Ag Fiber/PS	240	10	80%	[35]
Glass/AgNWs/OTS	130.5	20–250	88%	[36]
AgNWs/ZnO-TF/ZnO-NR	77.658	>100	80%	[39]
AGZO/AGZO/AGZO	180–240	2.5M	90%	[40]
Mutilayer Graphene/Graphene Scrolls	10–100	≈100	87–97%	[46]
MoO3/Graphene/MoO3	>15	200	85%	[47]
Graphene/AgNWs/Graphene	——	10.8	77.6%	[50]
ITO/NF-r-CA/AgNWs	——	24	90%	[54]
ITO/PEDOT:PSS/PET	150	40–60	60–90%	[55]

## Data Availability

All the data shown in this manuscript are well noted the reference, and no Data Availability Statement is required here.
